# A dashboard for the evaluation of the effect of school closures on wellbeing of children and parents

**DOI:** 10.1186/s13690-023-01114-2

**Published:** 2023-10-03

**Authors:** Febe Brackx, Bert De Smedt, Geert Molenberghs

**Affiliations:** 1 L-BioStat, KU Leuven, Louvain, Belgium; 2 Faculty of Psychology and Educational Sciences, KU Leuven, Louvain, Belgium

**Keywords:** COVID, Wellbeing, Dashboard, School, Lockdown, School closures

## Abstract

**Background:**

We present a dashboard for the evaluation of the impact of school closures on children and parents during the first wave of the COVID pandemic in 2020 on the various components of wellbeing.

**Methods:**

Starting from an explorative literature search by a team of experts from diverse fields (e.g., epidemiology, virology, psychology, education, sociology), we developed a dashboard that allows for the quick evaluation of the general effect of school closures on various indicators of well-being in different groups and for the quality of the available research, at a time where a crisis is ongoing.

**Results:**

It is concluded that there is evidence that the school closures reduced the transmission of COVID in the first wave in springtime 2020. Nevertheless, a multitude of studies show that the school closures also had a negative impact on different components of wellbeing such as academic achievement, time spent on learning and mental health. Furthermore, the school closures affected not only the children and adolescents, but also the parents that were forced to provide more childcare and help with schoolwork. Longitudinal studies on large representative samples with repeated assessments of wellbeing are necessary to understand the long-term effects of the school closures.

**Conclusions:**

The dashboard provides a first visual overview of the effects of school closures on wellbeing, and can serve as the basis for a future more systematic review and meta-analysis of the effects of school closures on wellbeing. It can be considered as a paradigm for rapid obtention of scientific evidence, during a quickly unfolding crisis, also in view of underpinning policy advice.

**Table:** 

Text box 1. Contributions to literature
• Our aim was to develop a dashboard for the evaluation of policy decisions at a point in a crisis when little or no information on the impact of these decisions was available.
• The current dashboard that summarizes the available evidence on the effect of school closures, provides a novel and integrative overview because it summarizes the effect of school closures on different components of wellbeing in different age groups.
• The dashboard serves as an example for a fast evaluation of policy decisions on a wide variety of indicators in crises where limited scientific studies are available.

## Introduction

During the COVID pandemic, a range of non-pharmaceutical interventions (NPIs) were implemented to reduce the number of infections, such as stay-at-home orders, school closures, closure of bars and restaurants, travel bans, etc. Many of the measures had an effect on the general wellbeing of the population affected by them. The KU Leuven Metaforum, an interdisciplinary think tank for societal debate, launched a working group on Pandemic Preparedness [1]. In 2021, a subgroup of experts was created to evaluate the effect of different NPIs on different components of wellbeing for different target groups [2]. In this paper, we focus on school closures during the first wave of COVID infections in spring 2020 and present a dashboard for the evaluation of their effects on wellbeing in the Belgian context. The aim of the dashboard is to provide a visual overview of the effect of school closures on different components of wellbeing and for different groups.

To evaluate the impact of school closures, the crucial first question is epidemiological in nature: Did the school closures have an impact on the number of infections, and was there a difference between primary schools, secondary schools and higher education? Furthermore, the effect of school closures on education is of importance. It is hypothesized by many that the school closures will lead to long term negative effects on the performance of the children and adolescents affected by them [3–5], while others predicted that negative effects on school performances can be mitigated or the school performances will return to normal after schools reopen [6]. As the pandemic started two years prior to the writing of this report, only short term effects of the school closures are currently known. Longitudinal studies are necessary to estimate the true long term effects. The components of mental health and work-life balance are included in the dashboard because they are important components of wellbeing, but as the school closures were enforced in combination with other NPIs (such as stay-at-home mandates, travel restrictions, teleworking mandates, closure of non-essential shops, etc.), it is very challenging to distinguish the effect of school closures on mental health and work-life-balance from the effects of other measures. Most studies that are included in the dashboard for these two components of wellbeing are therefore on the effect of the lockdown in general. The creation of a dashboard by academics with demonstrable research expertise can be considered as a useful methodology when there is urgent need for scientifically sound policy advice, but when there is little time for a conventional systematic review and when conducting such a review would call for a too lengthy process.

## Background

Wellbeing has been defined by [7] as the combination of feeling well (incorporating positive emotions of happiness, contentment, interest, engagement, confidence and affection) and functioning effectively (developing one’s potential, having a sense of purpose and control over one’s life and experiencing positive relationships). The Organisation for Economic Co-operation and Development (OECD) has provided a framework for wellbeing, consisting of 11 key dimensions and more than 80 indicators [8]. The dimensions are: income and wealth, work and job quality, housing, health, knowledge and skills, environment quality, subjective wellbeing, safety, work-life balance, social connections and civil engagement. Many studies are investigating the effect of the measures on wellbeing in isolation, focusing on one specific measure (such as school closures, [9]), one specific domain of wellbeing (such as sleep patterns and disturbances, [10]) and/or on one specific group of people (such as adolescent girls, [11]). However, every measure is expected to affect different dimensions of wellbeing, and in order to evaluate measures in a balanced way, as many as possible domains of wellbeing and all people affected by them should be considered together. School closures for example are expected to affect not only the number of infections, but also other wellbeing indicators such as the school performances of children and the work-life balance of the parents. This motivates the development of a dashboard for the effect of school closures, where different dimensions of wellbeing are considered together.

Many countries enforced school closures for several months during the first wave of COVID in springtime 2020, resulting in a global disruption of the education systems. One and a half billion school children, or 90% of the children worldwide, were affected by school closures [12]. The duration of the school closures ranged from a couple of weeks in some countries (e.g. Norway, Finland, Denmark) to more than a full school year in others (e.g. India, Uganda, Chile). The transition to distance learning also depended on the connectivity and availability of electronic devices at home. Therefore, the effects of school closures on wellbeing are expected to be widely different for different countries. Because the original aim of the interdisciplinary think tank from which this study derived was to focus on developing a dashboard for local policy makers, most studies that are included in the dashboard are based on data from countries for which the epidemiological, social, economic and educational context is to some extent comparable to the one in Belgium. This allowed us, from a practical point of view, to transfer the results to the Belgian context.

## Methods

The subgroup of academics^1^ was created in order to quickly scan the literature and summarize the results in a shared file. The goal was to represent a wide range of disciplines. This subgroup was created on short notice in the midst of the COVID pandemic including academics that had a proven track record in their discipline and that were available for this project. In retrospect not all relevant disciplines could be included, and for future work, we would recommend to include additional experts, for example on antropology, ethics and law. Each researcher focussed on a different component of wellbeing. For example, the component on phyiscal health was covered by the researchers in epidemiology and virology, the component of emotional wellbeing was covered by a researcher in psychology, and the component of education was covered by a researcher in educational sciences. There were no inclusion criteria, as the literature was emerging during the project and results were added to the shared file in a progressive way. Papers published in peer-review journals as well as government reports were considered. Only papers and reports published in English or Dutch were included. The keywords that were used depended on the component of wellbeing, but in most cases included “COVID” and “school”.

The rows of the dashboard consist of the target individuals or groups (e.g., 6-11-year-olds). The goal is to distinguish groups when it is expected that the effect of school closures will be differentiated and/or special weight could be given to these groups. For this dashboard, the evaluation is differentiated according to age, sex and socioeconomic status. Furthermore, three different work statusses are considered: people working in education, people having an essential occupation and people that are unemployed.

The columns of the dashboard consist of the dimensions of wellbeing, based on the OECD Better Life Index, each represented by specific indicators. The key dimensions by the OECD are adapted to the context of school closures. For example, Knowledge and Skills is translated to Education, and is represented by the indicators Achievement and Learning time. Furthermore, the components and indicators are determined through consensus by the Metaforum experts after thorough discussion, taking into account the availability of research on these indicators of wellbeing. The dimension of Environment quality for example was omitted, since there is no available literature on the effect of school closure on this component. The goal is to give a comprehensive picture of the components of wellbeing that can be affected by school closures. The components of wellbeing that were included in the dashboard are physical health, education, work-life balance, mental health or life satisfaction and income. The various components were approximated by specific indicators, such as infections and weight gain for physical health, and achievement and learning time for education.

**Fig. 1:**
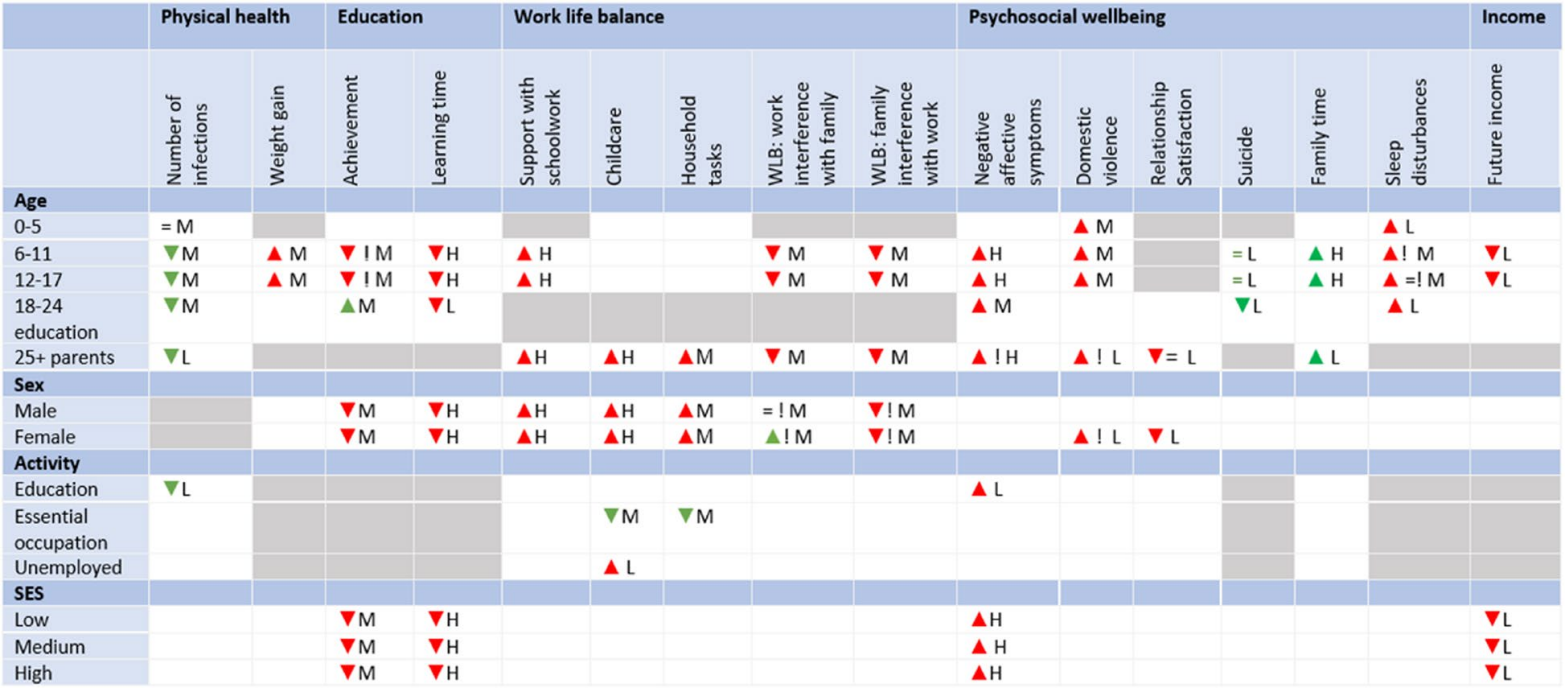
Dashboard for the effect of school closures during the COVID pandemic on different components of wellbeing in different groups. The general effect of school closures on an indicator of wellbeing are represented by arrows: ▲ (increase), = (no effect) or ▼ (decrease). If conflicting results were found in literature, arrows in both directions are included in the dashboard. The color of the arrow represents whether the effect is favorable (green) or unfavorable (red). An exclamation mark is added to the effect in case there are important exceptions or interaction effects. The areas that are deemed irrelevant are greyed out in the dashboard. The quality label “Low” (= L) is assigned if only limited research is available on the topic, and/or the research is of low quality (for example, when no comparison with a relevant baseline observation is performed). The quality label “Moderate” (= M) is assigned if there is one or more studies with strong methodological features or multiple studies that are not of high quality themselves, but are in combination (complementary strengths). Finally, the quality label “High” (= H) is assigned if there are multiple convincing studies with similar results. In this dashboard, SES stands for socio-economic status, and WLB stands for work-life balance

The results of the literature search are summarized in the dashboard, shown in Fig. 1. The general effect of school closures on an indicator of wellbeing are represented by arrows: ▲ (increase), = (no effect) or ▼ (decrease). If conflicting results were found in literature, arrows in both directions are included in the dashboard. The color of the arrow represents whether the effect is favorable (green) or unfavorable (red). An exclamation mark is added to the effect in case there are important exceptions or interaction effects. Results on differences between groups (such as an age effect) are not shown in the dashboard, but are described qualitatively in the results section. The areas that are deemed irrelevant are greyed out in the dashboard.

The quality of the evidence (low, moderate, high) is included in the dashboard for each cell. The assessment of the quality is based on different factors. Firstly, the methodological features of studies are taken into account: Is the study cross-sectional or longitudinal? Is baseline (or pre-pandemic) information available for comparison?

Are the results self-reported? What is the sample size? Is the sample representative (or is it based on self-selection)? Secondly, the consistency of the results is taken into account, with contradicting results from different studies lowering the quality of the evidence. Finally, the transferability of the results to the Belgian context is included in the evaluation, with results from neighboring countries (Germany, France, the Netherlands) valued higher. The quality label “Low” (=L) is assigned if only limited research is available on the topic, and/or the research is of low quality (for example, when no comparison with a relevant baseline observation is performed). The quality label “High” (=H) is assigned if there are multiple convincing studies with similar results. Finally, the quality label “Moderate” (=M) is assigned if there is one or more studies with strong methodological features or multiple studies that are not of high quality themselves, but are in combination (complementary strengths).

As the COVID pandemic is a recent phenomenon with progressively more evidence becoming available, a conventional systematic review was not the aim of this project. Speed was prioritized over perfection. The selected literature represents an opportunity sample of all available literature, and can be used as the basis for later more systematic research. The factors that are described above are used in a qualitative way, where the assigned quality label is based on the judgement of the researcher. A more standardized approach would be useful in a later systematic review, but was not part of this project.

## Results

The dashboard is shown in Fig. 1. The results are discussed for the different components of wellbeing separately. An overview of the publication years of the papers included in the dashboard for the 5 components of wellbeing is presented Table A1 in Appendix. An overview of the geographical context in which the papers included in the dashboard are published is presented Table A2 in Appendix. The regions are based on the United Nations geoscheme [13].

### Physical health

#### Number of infections

The first component of physical health is represented by the number of SARS-Cov-2 infections. Estimating the effect of school closures on infection rates is challenging, as school closures were generally implemented alongside other NPIs. Nevertheless, there is moderate evidence that in the first wave school closures contributed to a reduction in community transmission, hospital admissions and deaths. It is hypothesized that the effect of school closures on infection rates is driven by both the reduced contacts among children and the reduced contacts among adults as parents, who had to stay at home for child care.

A study from ECDC modelled the time-changing reproductive number as a function of school closures and other NPIs, taking into account between-country differences and time-changing effects. They concluded that the closure of secondary schools (which typically start at the age of 12 years) had the strongest impact on community transmission, followed by closure of higher education. The closure of primary schools and daycare centers were estimated to be less important to the reduction in community transmission [14]. This age gradient is also seen in other studies ([15, 16] for England, [17] for Belgium).

Secondly, the effect of school closures was higher in the first half of 2020, compared to the second half of 2020. This is partially caused by the implementation of physical distancing and hygiene measures in schools, protecting both educational staff and students from getting infected in school [14]. Furthermore, the effect of school closures is likely to depend on the COVID variant that is dominant at that moment, and the vaccination rate in the population at that time. In a study on primary school children in December 2020 in Liège, Belgium, transmission trees were reconstructed and it was shown that most of the transmissions occurred between children and between employees within the school, and spillover took place from infected children and teachers to their parents. It was concluded that most transmission events originated from within the school and the implementation of additional measures to reduce transmissions at school should be considered [18]. By September 2021, the vaccination coverage of adults was large in most European countries, while particularly children below the age of 12 had a very low vaccination coverage. Therefore, transmission in schools was more likely to be of concern in this period.

Furthermore, the effect of school closures depends on the effect of other NPIs implemented simultaneously. It was demonstrated that the additional benefit of school closures is low when other non-school-based measures, such as enforced teleworking or the closure of bars and restaurants, are not yet exhausted [15].

To conclude, the effect of school closures on COVID transmission in the general population is complex, as it depends on the COVID variant, the age of the individuals, other NPIs, vaccination coverage, school-related mitigation measures, etc.

#### Weight gain

There is moderate evidence of a negative effect of lockdown measures on body weight. Significant increases in body weight and BMI during lockdown among school-aged children and adolescents were observed, together with an increased prevalence of obesity and overweight. It is important to note that the effect on body weight is not only caused by school closures, but by all measures reducing physical activity and increasing sedentary behavior. A systematic review and meta-analysis is provided in [19], which indicates that young children were more affected than adolescents, as larger gains in weight and BMI were reported for younger children in different studies.

### Education

#### Achievement

During the first lockdown, many people raised concerns about the (potentially long-term) negative impact of school closures on the academic progress of children and adolescents [3–5]. Different sources of evidence on the academic performance during the pandemic are available. The sources differ in the design of the study (cross-sectional studies comparing the cohort of students affected by school closures in 2020 with older cohorts with the same amount of schooling in previous years, versus longitudinal studies comparing performance of the same group of students before the pandemic and in 2020), timing (during the first period of school closures, immediately after the first period of school closures at the end of school year 2019-2020, or in the beginning of school year 2020-2021), sampling method (national testing programs with a large representative sample versus small studies with a convenience sample) and measurement of academic performance (performance versus growth). Furthermore, the effect of school closures on the achievement of students is expected to depend on factors such as the duration of the school closures, the access to digital learning devices and the study program (for example, only repeating previously taught materials or focusing on basic skills of mathematics and language), which are all country-dependent and sometimes even region-dependent. It is therefore difficult to directly compare the results of the studies, but tentative conclusions can be drawn. These are discussed as a function of age, sex and socio-economic status (SES).

##### Age

For primary school children, mixed results are found, with declines, stability and even improvements compared to cohorts of previous years, dependent on the topic and the country. In a study in Flanders (Belgium), exactly the same standardized tests were administered to grade 6 (12-year-olds) of primary education in 2019 (pre-pandemic), 2020 and 2021. In the 2019-2020 school year, significant learning losses in language and mathematics were observed compared to the previous cohorts. [20]. A study in the Netherlands on 350,000 primary school children aged 7 to 10 showed significant learning losses on national exams after the lockdown based on a longitudinal difference-in-difference analysis [21]. Also in France, national standardized tests are performed for language and mathematics for students aged 11. A small increase was reported in the percentage of children with satisfactory or better mastery of language and mathematics [22]. A study in the state of Baden-Württemberg in Germany, based on statewide mandatory assessment of reading and mathematics competencies for grade 5 (age 11), reported a minor drop in the mean competence scores for arithmetic operations and numbers, and a minor increase in the mean competence scores for reading [23]. In both France and Germany, the assessments were performed in September 2020, in the beginning of school year 2020-2021. Important to note in both studies, similar fluctuations in performance have been reported in earlier years. One therefore has to be careful with causal conclusions and with attributing this decline to the effect of school closures. A study on more than 4800 children (age 8-9) in Australia compared achievements in 2020 to achievements in 2019 in a set of matched schools and reported no significant differences for mathematics or reading [24]. Another study from the UK reported significant reductions in growth for reading and mathematics [25]. Finally, in Italy, a study comparing mathematics achievements of the pre-covid and covid cohort in grade 3 concluded a large negative impact [26].

Fewer studies are available for secondary school students, yet one study from the UK reports a significant reduction in growth for reading for students up to Year 9 (age 14) [25]. A study from the Netherlands, making use of an online practice tool for studying the vocabulary of a foreign language, reported a minimal difference in students’ progress compared to previous years (students age 12 to 16) [27].

For students in higher education, a positive effect on the study performance is reported in different countries ([28] for Spain; [29] for Turkey; [30] for the USA). In Flanders, the performance of students at the universities and university colleges improved in the exam period of June 2020 compared to previous academic years [31].

##### Sex

Mixed results are found regarding the relationship between sex and learning losses. In the Netherlands, no significant sex difference in learning loss was found after the lockdown of 8 weeks [21]. In another Dutch study, the effects of sex differed by study domain: the difference in learning growth for spelling was larger for girls than boys, while the opposite was observed for mathematics. For reading, only a small difference between boys and girls was observed [32]. In Denmark, results from standardized tests on reading conducted between 2015 and 2021 on public school children aged 8 until 14, indicated that learning losses were larger for boys than girls [33]. In France, small improvements in achievements were reported, and these improvements were larger for boys than girls, thereby reducing the gender gap in study results [22].

##### Socio-economic status

Mixed results are reported regarding the socio-economic gradient. In Flanders, it was concluded that the attainment deficits were larger for schools that harbour more students with low educated mothers [20]. In the Netherlands, it was concluded that the decline in learning gains was larger for students from a low socioeconomic background [21]. On the other hand, the study in Baden-Württemberg in Germany reported only a minor effect of the average socio-cultural capital and the proportion of students with migration background on learning losses. Also in Denmark, little evidence was found of widened learning gaps by socioeconomic status [23, 33].

#### Learning time

A second indicator of the effect of school closure on the education, is the total amount of time children and adolescents spent on learning. In a report from OECD, data on home learning time during the first period of school closures is presented for the UK, France, Germany and Ireland. In all these countries and for all age categories studied (primary and secondary education), the total learning time has decreased compared to times without school closures, to about half of the usual instruction time at school in normal conditions [34]. There is evidence from Germany, France and the UK that during the period of school closure, girls spent more time on school work than boys ([35] for Germany, [9] for the UK, [22] for France).

##### Socio-economic status

For the countries for which survey data is available, mixed results were found for the association between the total learning time and the socio-economic gradient. In the UK, total study time was positively related to the education level and income of the parents [25]. In France, children from disadvantaged households spent less time on schoolwork [22]. In the United States, a positive association was found between time spent on schoolwork and education level of the parents, but no association with the household income or ethnicity. In Germany, only a weak association with education level of the parents was found, and this association was already present before the school closures [23]. Other studies report on the socio-economic gradient with respect to time spent on school work, and here as well there are mixed results (no significant effect of SES in a Swiss study [36], a significant effect of SES in a study from the UK [37]). Note that in most studies, no baseline measurement was available from before the pandemic, and therefore one has to be careful with drawing causal conclusions from this literature.

### Work-life balance

#### Support with school work

Different nationwide studies report an increase of time spent on parental support with school work. In France (for secondary school students) and Italy (for children < 14 years), two-thirds of parents reported to have spent more time than usual on support with school work [22, 38, 39]. In Germany, during lockdown the average time spent on support with schoolwork doubled [35, 40]. In the UK, parents on average spent 40 min more per day on childcare, including support with schoolwork, compared to the baseline observations [41]. Also in Flanders, the average time invested in support with school work increased [42, 43].

##### Age

The increase in time spent on support with school work depends on the age of the child, with parents devoting more time to younger children [22, 41–43]. It is likely that the age gradient reflects the greater independence of older children, combined with parents being less knowledgeable on the material taught in higher years.

##### Sex

Various studies indicate that mothers spent more time on support with school work than fathers during the periods of school closure ([22] for France; [44] for the United Kingdom; [45] for Germany [39] for Italy). This observation is consistent with the sex differences in household labor that were already present before the pandemic.

#### Socio-economic status

Some studies report a positive correlation between parental SES and support with school work ([22] for France; [40] for Germany; [39]for Italy), other studies report no association or a negative association ([46] for the UK; [47] for the USA). In a report from the OECD, it was hypothesized that the lack of consistent results is caused by the fact that people with high SES often worked from home during lockdown, while people with low SES were more often (temporarily) unemployed [34]. These groups therefore both had time to assist with schoolwork.

#### Childcare

Several studies from different countries concluded that the total time spent on childcare increased during periods of school closures ([48] for Hungary; [49] for the Netherlands; [50] for Spain; [37] and [51] for the UK).

##### Sex

Mixed results are found about the gender gap in childcare work during the lockdown. Several studies concluded that both mothers and fathers increased the time spent on childcare during lockdown, but the gender gap, with mothers spending more time on childcare than fathers, did not reduce. One study from the UK reports on the impact of the lockdown measures by using the latest data from the 2020 UK Household Longitudinal Survey (UKHLS) COVID study. The weekly childcare hours among those with children younger than 16 provided by the same individuals during and before (2015) the lockdown were compared. Also in this study, the results showed an increase in total time spent on childcare during the lockdown. Furthermore, in both periods, women spent approximately twice as much time as men on childcare. Therefore, this study concludes no increased or reduced gender gap in time spent on childcare [51]. Two other studies reported a slightly increased but nonsignificant relative participation in childcare by men ([50] for Spain; [37] for the UK). A study from the Netherlands reported a significant increase in relative share of the fathers in childcare work [49]. In another study from Australia, the increases in childcare were proportionally higher in fathers compared to mothers, resulting in a narrowed relative gender gap [52].

##### Socio-economic status

With respect to the socio-economic gradient, mixed results are found. In a study from the Netherlands, no significant difference was found in the probability of increasing or decreasing the relative share of care tasks during the first lockdown by the parent’s educational level [49]. On the other hand, case studies from Hungary and Italy showed that parents with a higher level of education spent more time on childcare tasks ([48] for Hungary, [39] for Italy).

#### Household tasks

For household tasks, similar results are found as for childcare. Several studies showed that the total time spent on household tasks (cleaning, cooking, etc.) increased during lockdown ([51] and [53] for the UK; [39] for Italy; [49] for the Netherlands; [50] for Spain). Mixed results are found for the gender gap in household tasks, with some studies reporting a reduced gender gap ([49] for the Netherlands; [53] for the UK), and other studies reporting no change in gender gap ([51] for the UK; [50] for Spain).

#### Work interference with family and family interference with work

For the general population, teleworking during the lockdown was associated with less work interference with family, and more family interference with work. Furthermore, both work interference with family and family interference with work were more often experienced by people with children than people without. Also, the age of children played a role: Parents of primary school children experienced both types of interference more than parents of secondary-school children. These conclusions were reached by a study across 27 European countries based on Eurofound’s Living, Working and COVID-19 survey data from April 2020 [54]. Difficulties with work-life balance for parents of primary school children were also reported by a study in Canada [55], the Netherlands [49] and the UK [56].

Working from home in combination with school closures improved work interference with family only among women, while it deteriorated family interference with work among both men and women. Especially teleworking mothers were more likely to experience family interference with work than teleworking fathers ([56] for the UK; [54] for 27 European countries; [55] for Canada). On the other hand, in a study from the Netherlands, no significant sex difference was found in the probability of facing an improved or deteriorated work-life balance during the first lockdown compared to before [49].

### Psychosocial wellbeing

The component of psycho-social wellbeing is represented by 7 indicators: the prevalence of negative affective symptoms, the prevalence of domestic violence, relationship satisfaction, suicide rates, family time and the prevalence of sleep disturbance. Important to note is that the studies of mental health during the pandemic are generally not specifically on the effect of school closures in particular, but on the effect of all non-therapeutic interventions in general. Therefore, the effect of school closures on the mental health cannot be seen separately from the effect of reduced social and extracurricular activities.

#### Negative affective symptoms

There is high evidence that the lockdown led to a deterioration of mental health (negative feelings, depressive symptoms, anxiety, worries) in children and adolescents. In a systematic review on the mental health impacts of the covid pandemic on children and adolescents, it was concluded that most studies observed an increase in the number of depressive and anxious symptoms and a worsening trend in general mental health, compared to before the pandemic. The effect was stronger for older children and adolescents, girls, and children and adolescents living with neurodiversities and/or chronic physical conditions [57]. Another systematic reviewconcluded that school closures harm the wellbeing of children and adolescents [58]. Since the publication of these two reviews, other relevant studies have been published.

One study from the Netherlands compared the mental and social health of a representative sample of children and adolescents from the general population during the COVID-19 lockdown to a similar sample of children and adolescents before COVID-19. Children and adolescents reported poorer mental and social health during the COVID-19 lockdown on all six PROMIS domains: global health (i.e., the general, physical, mental, and social health of an individual), peer relationships, anxiety, depressive symptoms, anger, sleep-related problems [59]. In a longitudinal German study on the wellbeing of children and adolescents, it was found that scores in psychological wellbeing were significantly lower during lockdown compared to baseline (pre-pandemic measurement in 2019). The effect was significantly stronger in children with a medium to low socio-economic status. Furthermore, girls experienced more anxiety than boys [60]. This socio-economic gradient is also reported in other studies ([61] for England).

Although less studied, it is shown that also students in higher education were affected by the school closures. In a systematic review including 16 studies on higher education students, it was found that the overall mental health of students worsened compared to pre-pandemic times [62].

School closures not only affect the students, but also the parents. It has been shown that the mental health of the general population has deteriorated during the first lockdown, but somestudies have shown that the mental health of parents deteriorated even more ([63] for Canada, [64] for France). Both these studies also report that the deterioration of mental health was worse in single parents compared to couples with children.

#### Domestic violence

Although a decrease in police reports and referrals to child protective services is reported, different studies show an increase in child abuse-related injuries treated in hospitals, and an increase in family violence reported in surveys, indicating that the closure of schools and childcare settings may have considerable negative consequences for children in vulnerable situations [65–67]. Furthermore, there is some evidence that the presence of children at home is a risk factor for violence against mothers [68, 69].

#### Relationship satisfaction

Mixed results were found for the effect of school closures on the relationship satisfaction of parents. A negative effect, positive effect and no effect of having children on the relationship satisfaction were reported. A Japanese study on the mental health of mothers of school children concluded that the relationship satisfaction decreased during school closures [69]. A German study concluded that the relationship satisfaction deteriorated more in couples without children than in couples with children [70]. A Dutch survey concluded that the majority of parents report no deterioration in the relationship dynamics [49]. Finally, a longitudinal study from the USA reported that the presence of children did not moderate the change in relationship satisfaction over time [71].

#### Suicide

No evidence was found that the suicide rate among adolescents was higher during the first wave of COVID, as reported by [58]. In contrast, two studies report an increased suicide rate among children and adolescents in the second wave, corresponding to the period after the end of the school closures. It is hypothesized that this is caused by an increased academic stress related to schools reopening, in combination with continued social distance measures leading to isolation [11, 72].

#### Family time

In different nationwide studies, it was reported that the time parents spent with their children has increased during the first wave of covid. In Canada, 65% of parents reported spending more quality time with their children [63]. In France, the percentage of parents of secondary school students reporting spending more, less or as much time as usual on leisure activities is 40%, 28% and 25%, respectively [22]. In Switzerland, 73% of parents agreed on school closures being an opportunity to spend more quality time with their children [73].

#### Sleep disturbances

Various studies indicate that the average sleep duration for children and adolescents has increased during the first covid wave [10, 74, 75]. Some studies indicate an increased level of sleep disturbances, such as difficulty falling asleep and nightmares, especially for the younger age categories [10, 58, 74, 76, 77]. For adolescents, mixed results are found. A study from the UK reported that the quality of sleep deteriorated more for the older secondary school students compared to other age groups. The study hypothesized that this is caused by the public examinations taking place in those years. In this study, a strong association was found between deteriorated sleep quality and interpersonal functioning during the lockdown. Furthermore, the quality of sleep is more affected in girls compared to boys [74]. In contrast, an Italian study reports that the quality of sleep is the least affected in adolescents [10].

### Future income

Some simulation studies showed a decrease in future income for students that are now affected by school closures [78–81]. These studies are based on very strong assumptions (such as no catch-up over time), and currently the long-term effects of school closures are not known. Longitudinal studies are needed to estimate the effect of the school closures on the income of students affected by them.

## Discussion

There is evidence that the school closures reduced the transmission of COVID in the first wave in springtime 2020. The closure of secondary schools and higher education was of more importance for this effect than the closure of primary schools and daycare centers.

Studies on academic achievement after the first lockdown led to mixed results. For primary and secondary school children, declines, stability and improvements were found for different topics and countries. In Flanders specifically, significant learning losses in language and mathematics were observed by the end of school year 2019-2020 compared to previous cohorts. During school year 2020-2021, the teachers, students and parents were better adapted to remote learning. It is therefore expected that the effects of the COVID-related school closures on achievement are less severe compared to the first lockdown.

Some studies have been performed on the achievement of students at the end of school year 2020-2021. At that moment, students had experienced the initial school closure, and a full school year of learning interrupted by school closures and partial distance learning. In Flanders, the same standardized tests were administered to grade 6 of primary education in 2019, 2020 and 2021. The resilience in school outcomes at the end of school year 2020-2021 differed per subject. Additional declines were observed for language and French, while for math no additional deficits are observed. For science, the catch up process compared to previous cohorts seems to have started [82]. In a study from the Netherlands, a lower learning growth was observed for spelling, reading and mathematics, based on standardized test scores administered at the end of school year 2020-2021 in primary education [32]. A Danish study based on standardized test results on reading from children aged 8 until 14 concluded that the initial school closures led to learning losses, but that these learning losses did not increase during the subsequent school year [33]. An Italian study based on standardized tests performed in primary and secondary schools reported mixed results: decreases as well as increases were observed in performances dependent on the topic [83]. Further longitudinal studies are needed to identify the effects of the first lockdown and later measures on the achievements of students on the long term.

For the component of work-life balance, it can be concluded that parents spent more time on support with school work during periods of school closures. The increase is larger for younger children and for mothers. Also, the time spent on childcare and household tasks increased. Furthermore, both work interference with family and family interference with work were more often experienced by people with children than people without children.

Although there is strong evidence that the mental health of the general population, but also of children and adolescents, deteriorated during the initial lockdown in spring 2020, the impact of the COVID pandemic beyond the first lockdown is complex, and is associated with the tightening of measures. Longitudinal studies on large representative samples with repeated assessments of mental health are necessary to understand the long-term effects.

Very limited longitudinal studies with repeated assessments of mental health before and during the pandemic are available. One of them is the CO-SPACE study in England, tracking the mental health of children and adolescents age 4-16 throughout the pandemic using monthly surveys. It is reported that together with the easing restrictions from February to April 2021, the behavioral, emotional, and attentional difficulties decreased, especially for primary school children. Nevertheless, children from low-income households and children with special educational needs and/or neurodevelopmental disorders continued to show decreased mental health, and therefore did not show this post-lockdown recovery [61].

In Belgium, the Mental Assessment Group (MAG) reports on the evolution of the mental health state of the Belgian population throughout the pandemic. Supported by data recorded within the unit for adolescents of the Hospital Centre le Domaine-ULB in Braine-l'Alleud, a peak is reported in the child psychiatric mental health care system in January 2021, indicating a wave of psychological decompensation among young people (period of deterioration of a person’s existing mental health disorder). A second peak was reported in May 2021, corresponding to students returning to school full time [84].

From the dashboard, it is clear that not every component of wellbeing is well studied for all the defined groups. One example is the socio-economic gradient for most components of mental health. Also, young children (<5 years) are not well studied and very little is known on the effect of the pandemic on their development.

Furthermore, most studies that are included in the dashboard are on a population from another country, as very limited studies are performed in Belgium. In case of the achievement for example, only one study is published on Flemish children, and this study is limited to children from grade 6 in the Catholic schools, as this is the group where centralized tests already were implemented pre-COVID [20]. A more widely adopted central testing system would enable the longitudinal follow-up of children and adolescents affected by school closures. As the extrapolation of results from other countries to Belgium is not straightforward, more research conducted in Belgium on the effects of school closures on wellbeing would be helpful.

The quality of research is the highest for the number of infections and school achievement, as this research is based on government reported numbers and standardized tests that were already in place before the pandemic. On the other hand, studies on mental health and work-life balance are often based on self-reported data and self-selected samples, and are therefore more prone to bias. Indeed, a lot of studies are based on convenience samples with a bias to people with mid-and high socioeconomic status. Future studies should therefore specifically reach out to vulnerable groups, such as individuals from low-income families, persons with a migration background, etc. In [85], another study by the Metaforum working group on Pandemic Preparedness, the experiences and social affordances of informal solidarity in Belgium is summarized for invisible groups who are likely to experience structural and incidental forms of precarity during the pandemic and who are under-represented in surveys and administrative data.

A multidimensional, balanced evaluation of policy interventions is crucial. Nevertheless, current policy discussions are inclided to investigate different dimensions of wellbeing in isolation. We have collected the necessary information for a coherent evaluation, considering the various dimensions together.

Although the dashboard provides a useful visual overview of the effects of school closures on wellbeing, the lack of a standardized approach for the literature search and assignment of a quality label is a limitation. At the moment, the existing body of evidence is too small to perform a reliable systematic review. Once the evidence will have accumulated further, it would be valuable to perform a systematic review and a more standardised assessment and summary of study results.

## Conclusions

In this project, an overview is given of the literature on the effect of school closures during the first wave of the COVID pandemic in 2020 on the different components of wellbeing. An explorative literature search was performed by a team of experts from different fields, and the results were summarized in a dashboard. The dashboard provides a visual overview of the effect of school closures on different components of wellbeing and for different groups, enabling a multidimensional evaluation. This is particularly relevant during a crisis, when decisions have to be made very quikly while at the same tiem, immediate actie is required.

It can be concluded that there is evidence that the school closures of particularly secondary and higher education reduced the transmission of COVID in the first wave in springtime 2020. A multitude of studies showed effects of school closures, albeit with mixed results, on academic achievement. The closures also had a negative impact on different components of wellbeing, for example a deterioration of mental health The school closures additionally affected the parents, who were forced to provide more childcare and help with schoolwork. As children and adolescents suffer much less from severe COVID or COVID-related death compared to other age groups and the impact of school closures on the lives of children is enormous, a broad consensus is that school closures should serve as a measure of last resort. A dashboard crafted by recognized experts can be a valuable tool to underpin policy advice in the midst of a rapidly unfolding crisis, when there is little time for a conventional systematic review and when there are still important gaps in scientific knowledge because of the novel nature of the threat that creates the crisis, as was the case with SARS-CoV-2.

## Data Availability

Data sharing is not applicable to this article as no datasets were generated or analysed during the current study.

## Appendix

**Table A1:** Overview of the publication year of the papers included in the dashboard for the 5 components of wellbeing

**Component**	**2020**	**2021**	**2022**
**Physical health**	0	5	0
**Education**	8	10	2
**Work-life balance**	18	4	0
**Psychosocial wellbeing**	5	12	6
**Income**	1	2	1

**Table A2:** Overview of the geographical context in which the papers included in the dashboard were pusblished, for the 5 different components of wellbeing. The regions are based on the United Nations geoscheme

Component	Western Europe	Northern Europe	Southern Europe	Eastern Europe	North-America	Australia	Other
**Physical health**	4	0	0	0	0	0	0
**Education**	12	1	3	0	1	1	1
**Work-life balance**	13	0	1	1	2	1	0
**Psychosocial wellbeing**	9	1	2	0	2	0	3

## Footnotes

1 The team of academics consisted of: Sarah Delcourt (economics and business), Bert De Smedt (educational sciences and psychology), Leen Delang (virology), Siegfried Dewitte (economics and business), Florian Lange (behavioral economics), Geert Molenberghs (biostatistics & epidemiology), Erik Schokkaert (head of Metaforum project), Elien Smeets (virology), Thérèse Steenberghen (geography), Sem Vandekerckhove (social sciences on work, organisation and social dialogue), Geert Van Hootegem (sociology), Wim Van Lancker (sociology), and Julie Vinck (sociology).
